# Management of primary sclerosing cholangitis and its complications: an algorithmic approach

**DOI:** 10.1007/s12072-020-10118-x

**Published:** 2020-12-30

**Authors:** Michal Prokopič, Ulrich Beuers

**Affiliations:** 1 Department of Gastroenterology and Hepatology and Tytgat Institute for Liver and Intestinal Research, Amsterdam University Medical Centers, Location AMC, AGEM, C2-327, Meibergdreef 9, 1100 DE Amsterdam, The Netherlands; 2 Department of Gastroenterology, Comenius University Bratislava, Jessenius Faculty of Medicine, Martin, Slovakia

**Keywords:** Primary sclerosing cholangitis, Cholangiocarcinoma, Cholestasis, Bile acids, Pruritus, Ursodeoxycholic acid, Obeticholic acid, FXR, Bezafibrate, Liver transplantation

## Abstract

Primary sclerosing cholangitis (PSC) is a rare cholestatic liver disease, characterized by multiple strictures and dilatations of the intra- and extrahepatic bile ducts, leading to progressive liver fibrosis, in 10–15% cholangiocarcinoma, and ultimately end-stage liver disease. The pathogenesis is poorly understood, but (epi-)genetic factors, mechanisms of innate and adaptive immunity, toxic effects of hydrophobic bile acids, and possibly intestinal dysbiosis appear to be involved. The strong link with inflammatory bowel disease (IBD) is associated with a markedly enhanced risk of colorectal cancer which next to cholangiocarcinoma represents the most serious diagnostic challenge in long-term PSC management. Despite extensive research, no medical treatment has been proven so far to prolong the time to liver transplantation (LTx), which remains the effective treatment in late-stage disease. Recurrence of PSC after LTx is observed in up to 20% of patients. Here, we briefly summarize actual views on PSC pathogenesis and provide an algorithmic approach to diagnostic procedures and recommendations for the management of PSC and its complications. We describe promising treatment options subject to current clinical trials.

## Introduction

Primary sclerosing cholangitis (PSC) is a chronic cholestatic hepatobiliary disease of unknown etiology affecting both intra- and extrahepatic bile ducts. PSC is characterized by multifocal fibrotic bile duct strictures and dilatations with cholestasis, the gradual development of biliary liver fibrosis and cirrhosis, and deterioration of liver functions requiring liver transplantation (LTx) [1]. The disease can be asymptomatic for a long time, but a large proportion of patients may develop symptoms of pruritus, fatigue, right upper quadrant (RUQ) abdominal pain and fever as a sign of bacterial cholangitis, or symptoms of concomitant inflammatory bowel disease (IBD) [2]. In some cases, the disease is diagnosed as a result of clinical signs and symptoms of cholangiocarcinoma, the most commonly associated hepatobiliary malignancy [3]. Despite extensive research, the management of PSC patients remains difficult, mainly due to insufficient pharmacological treatment options that would prevent the formation of strictures, progression of fibrosis, and development of malignancies. Still, several new drugs are under investigation for combined medical treatment representing the potential to improve survival and clinical outcomes in PSC [4].

## Epidemiology

PSC is a rare disease with an incidence of 0–1.3 per 100.000 inhabitants/year and a prevalence of up to 16 per 100.000 [5, 6]. Transplant-free survival of median 21.3 years was reported by a large population-based study from the Netherlands, where the vast majority was treated with ursodeoxycholic acid (UDCA, 92%); lower transplant-free survival times were reported elsewhere [7]. Mean age at diagnosis is around 35–40 years and men are affected more frequently [7]. Concomitant inflammatory bowel diseases (IBD) can be found in more than two-thirds of PSC patients, predominantly presenting with features of ulcerative (pan)colitis [8]. This association seems to be weaker (34%) in Eastern countries [9].

## Pathogenesis

Genetic factors contribute about 10% to a predisposition for PSC [10] and may explain the increased risk of PSC in first-degree relatives of PSC patients [11]. More than 20 risk genes predominantly within the HLA complex or associated with IBD and other immune-mediated diseases were identified in PSC patients in a large genome-wide association study [12].

These data confirmed the role of the adaptive immune system in the pathogenesis of PSC, as HLA classes I and II are involved in recognition of exogenous and endogenous antigens and their presentation to intestinal T-cells, which may be aberrantly homing in the liver in PSC with IBD [13]. The role of T-cells (Th_17_) has been confirmed in PSC-IBD patients in whom specific intestinal bacteria-induced pore formation in the intestinal epithelium and subsequent bacterial translocation with the immune-inflammatory response of the hepatobiliary tract [14]. Together with the recent finding of alteration in intestinal fungi composition, this reinforces the long-considered hypothesis of a causal role for gut microbiota dysbiosis in the pathogenesis of PSC [15].

Cholangiocytes respond to the recognition of environmental insults such as microbes, xenobiotics or bile acid-induced damage by triggering profibrotic and pro-inflammatory pathways. The “activated cholangiocyte” secretes proinflammatory cyto- and chemokines and may, thereby, ensure repair processes by activating cells of innate (macrophages) and adaptive (T-cells) immunity. During the chronic injury, however, it can lead to cholangiocyte senescence and differentiation of matrix depositing myofibroblasts from hepatic stellate cells and portal myofibroblasts resulting in tissue scarring and bile duct strictures [16].

The main mechanisms of cholangiocyte protection against the potentially harmful effects of biliary bile acid monomers are apparently the secretion of bicarbonate with the formation of a “biliary bicarbonate umbrella” [17], an alkaline layer on the apical membrane of cholangiocytes stabilized by the biliary glycocalyx [18], and the luminal formation of mixed micelles of phospholipids, cholesterol and bile acids. The “biliary bicarbonate umbrella” is supposed to sustain luminal hydrophobic bile acid monomers in a polar deprotonated state as bile salts and, thereby, prevent their uncontrolled, carrier-independent passage through the apical cholangiocyte membrane into the cell [18]. It is hypothesized that stabilization of the biliary bicarbonate umbrella may slow down the progression of fibrosing cholangiopathies [17, 18]. A role of the bicarbonate umbrella in PSC pathogenesis is supported by the association of PSC with gene sequence variations of TGR5, a cholangiocellular bile acid receptor promoting chloride and bicarbonate secretion [10], and by downregulation of the TGR5 protein in cholangiocytes of PSC patients [10, 19]. Also associations with PSC of other gene variants encoding for stabilizers of the apical cholangiocyte membrane of cholangiocytes are suggestive of defects of the biliary bicarbonate umbrella in PSC [10].

Intracellular accumulation of potentially toxic bile acids during cholestasis in humans contributes to hepatocyte and cholangiocyte damage, inflammation, and the development and progression of cholestatic diseases. One of the key mechanisms protecting hepatocytes against bile acid accumulation is the negative feedback regulation of their hepatic synthesis mediated by fibroblast growth factor 19 (FGF19), an endocrine hormone produced mainly in the ileum after farnesoid-X receptor (FXR) activation by bile acids [20]. Aberrant hepatic FGF19 expression was observed in liver explants of PSC patients, but not healthy controls, mirroring pathological accumulation of bile acids in livers of PSC patients [21].

## Diagnostic approach

### Clinical presentation

A majority of PSC patients are asymptomatic at the time of diagnosis [2, 22]. Nevertheless, it is possible to define several symptoms that may occur in the early stages of the disease representing mostly signs of complications of the disease. Abdominal pain in the right upper quadrant, often recurrent and sometimes associated with fever and chills or even jaundice, is one of the most common symptoms and may be a sign of bacterial cholangitis [23]. Pruritus of varying severity predominantly affecting the limbs is found independently of disease activity and occurs in more than two-thirds of patients during their lifetime [24]. It may indicate the presence of one or more major bile duct strictures. Fatigue is also quite frequently reported in PSC, but like pruritus, it is not associated with the severity of the disease [25]. Signs and symptoms associated with portal hypertension and decompensated liver cirrhosis (jaundice, hepatomegaly, splenomegaly, ascites, variceal bleeding) are rarely seen in the early stages but may manifest later in the disease course. Osteoporosis is associated with advanced PSC, but also with duration of IBD [26]. In prolonged severe cholestasis fat and fat-soluble vitamin malabsorption may occur, presented by steatorrhea, unintended weight-loss, and coagulopathy [2]. In Table 1, the most common symptoms described in various cohort studies are summarized with respect to their occurrence. Differences may at least in part be explained by differences in disease stages of the cohorts.

**Table 1:** Most common clinical symptoms in PSC in cohort studies

Study	Year	No. of patients	Abdominal pain (%)	Jaundice (%)	Cholangitis (%)	Pruritus (%)	Fever (%)	Fatigue (%)
Wiesner et al*.* [77]	1989	174	NR	59	28	59	NR	66
Broome et al*.* [78]	1996	305	37	30	NR	30	17	NR
Kaplan et al*.* [79]	2007	49	20	6	NR	10	4	6
Guerra et al*.* [22]	2019	277	8	8	5	11	NR	NR

*NR *not reported

### Serum markers

**Fig. 1:**
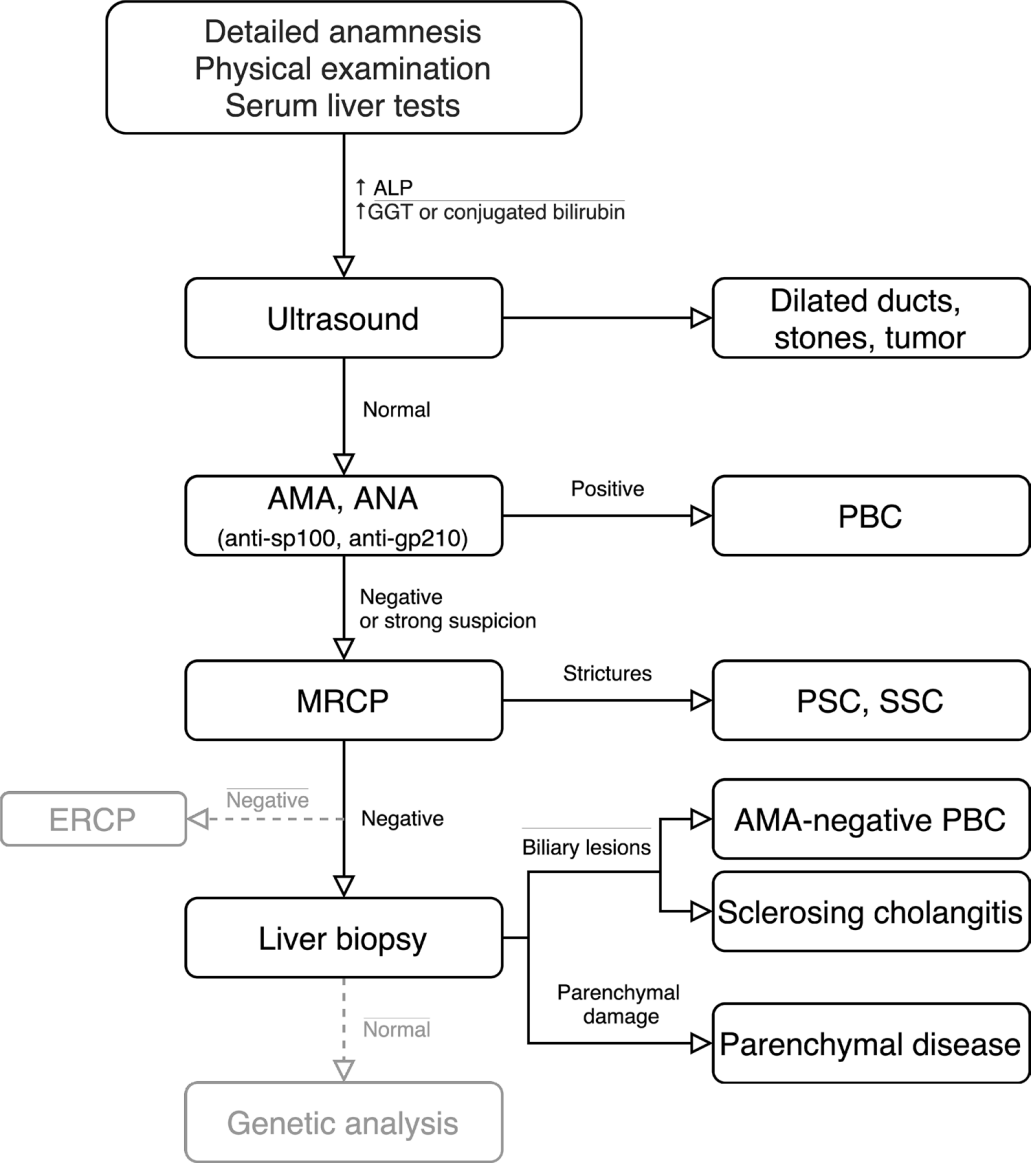
The algorithmic approach to the patient with cholestasis [2, 60]. *AMA* anti-mitochondrial antibodies, *ANA* antinuclear antibodies, *sp100* sp100 nuclear antigen, *gp210* glycoprotein 210, *ALP* alkaline phosphatase, *GGT* gamma-glutamyl transferase, *ERCP* endoscopic retrograde cholangiopancreatography, *MRCP* magnetic resonance cholangiopancreatography, *PBC* primary biliary cholangitis, *PSC* primary sclerosing cholangitis

Elevated serum levels of markers of cholestasis (ALP, γGT, conjugated bilrubin), particularly in patients with IBD, are often the first detected biochemical sign of PSC. Although elevated serum ALP is included in the diagnostic criteria for PSC [2], it may in some cases be within normal range at the time of diagnosis, as ALP levels fluctuate during the course of the disease [1]. Serum levels of transaminases (AST, ALT) are also often mildly elevated in patients with PSC, but a marked and persistent increase may indicate features of autoimmune hepatitis in PSC. Elevated conjugated bilirubin levels may be indicative of dominant bile duct strictures or more advanced disease [27]. IgG and IgM immunoglobulins may exceed normal limits in more than 50% of patients, yet are not specific for PSC. Autoantibodies (atypical pANCA, ANA, ASMA) may be also positive in a large proportion of patients [28], but their routine analysis is not necessary for the diagnosis of PSC due to their low specificity [2]. The proof of negative AMA and PBC-specific ANA (sp100, gp210) may help to exclude primary biliary cholangitis [29]. The algorithm for the diagnostic approach to the patient with cholestasis is presented in Fig. 1.

#### Imaging

**Fig. 2:**
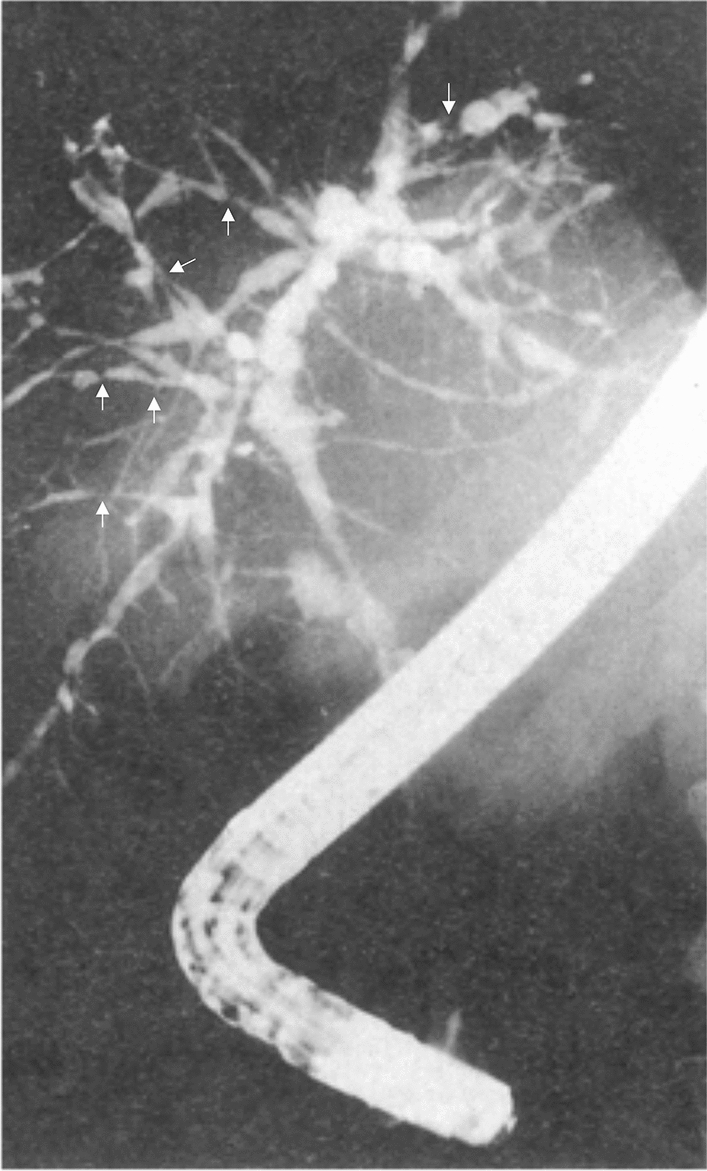
Typical cholangiogram in PSC. Multiple short strictures (indicated by arrows) and dilatations of intra- and extrahepatic bile ducts forming a “beaded pattern” are visible representing the characteristic ERCP finding in a PSC patient

Imaging studies are an essential part of the diagnostic process in a patient with cholestasis. Ultrasonography**,** which is usually the first imaging method performed in a patient with cholestasis, finds use also in diagnostics of sclerosing cholangitis by the exclusion of some causes of secondary sclerosing cholangitis (SSC) and recognition of possible gallbladder disease (stones, polyps, enlargement or wall-thickening) [30] or visualization of dilated bile ducts in some PSC patients. Nevertheless, magnetic resonance cholangiography (MRC) is the primary diagnostic imaging modality in patients with suspected PSC and should be performed and interpreted in experienced centers [31]. A typical cholangiogram in PSC shows irregular narrowing of bile ducts with multifocal short annular intra- and/or extrahepatic strictures alternating with slightly dilated segments, creating a “beaded” pattern (Fig. 2) [2]. ERCP should only be reserved for diagnostic cholangiography in patients with higher clinical suspicion of PSC in whom MRC is contraindicated, or when MRC and liver biopsy are ambiguous [32]. MRC can also be used to screen for PSC-associated malignancies and MR elastography (MRE) for non-invasive liver stiffness measurement to assess the stage of liver fibrosis [31]. Like MRE, more available and much more affordable shear-wave-based transient elastography correlates with the stage of fibrosis and outcomes in PSC and may be used for stratification of patients [31].

### PSC variants: time for a liver biopsy

**Fig. 3:**
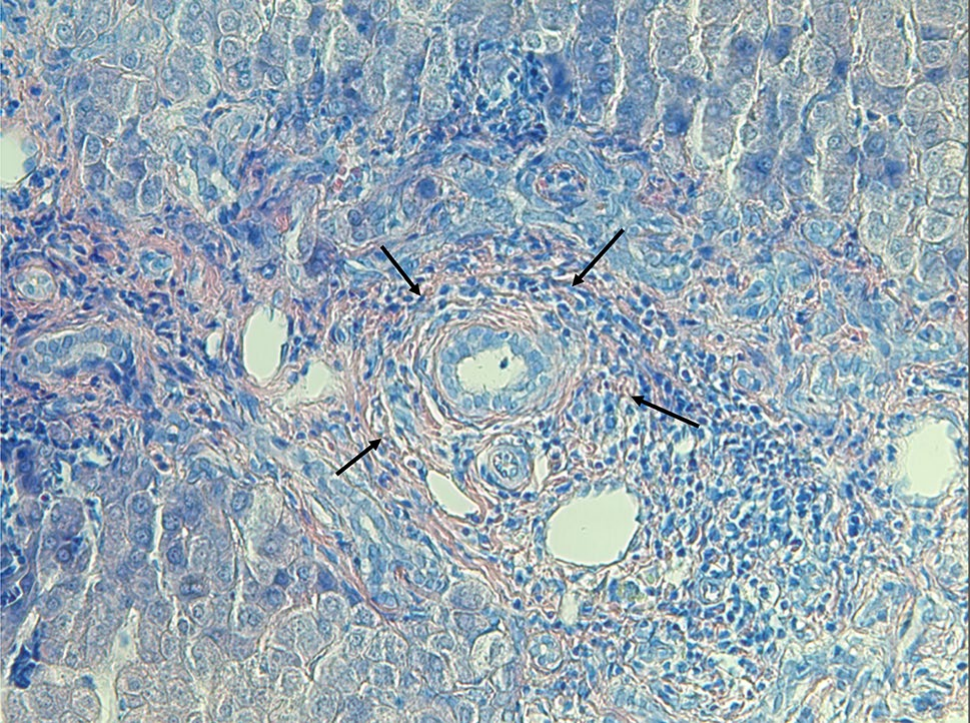
Histological findings in PSC. Concentric periductal fibrosis (“onion-skin”, indicated by arrows) with oedema and inflammatory portal cell infiltrate (Giemsa stain). The biopsy was performed on a 19-year-old man with newly diagnosed ulcerative colitis, markedly elevated cholestatic serum markers, and no cholangiographic changes. Five years later cholangiography showed typical findings of PSC

A histological finding characteristic, but not specific for PSC is an “onion-skin” pattern mimicking concentric periductal fibrosis with lymphocyte infiltration and portal edema (Fig. 3). To determine the stage of PSC, assess the disease progression, and predict the long-term outcomes and transplant-free survival, standard histological scoring systems are used (Tables 2, 3) [33–35]. Liver biopsy, especially due to its invasiveness and risk of complications, is not required for the diagnosis of PSC, however, in some cases remains irreplaceable [2]. Suspected small-duct PSC or PSC with features of autoimmune hepatitis (AIH) are conditions where a liver biopsy may be used to confirm or refute the diagnosis and indicate further management. Small-duct PSC, defined as a variant of PSC with clinical, biochemical, and histological features of PSC in the presence of a normal cholangiogram, can be found in around 5% of all PSC patients and represents a variant of PSC with better outcomes [36]. In a recent long-term follow-up study, 55% of small-duct PSC patients developed cholangiographic changes diagnostic of a large-duct PSC over time, supporting the hypothesis, that small-duct PSC is an early stage of a classical large-duct disease [37]. PSC with features of AIH occurs in approximately 7–14% of PSC patients [38]. Since the elevation of serum markers (transaminases, IgG, autoantibodies) may be present in both conditions, a liver biopsy is necessary to clearly determine a definitive diagnosis, quantitate the extent of hepatic inflammation and determine the treatment. It is recommended to treat PSC and AIH as if they were two separate diseases and therefore the management of AIH should follow the guideline for the treatment of AIH [38]. However, immunosuppressants that have been tested to date have not been successful in the treatment of PSC and are therefore not recommended unless the presence of features of AIH is shown [29].

**Table 2:** Nakanuma’s staging system [35]

Nakanuma’s staging system	
SCORING	
Score	Fibrosis
0	No portal fibrosis or fibrosis limited to portal tracts
1	Portal fibrosis with periportal fibrosis or incomplete septal fibrosis
2	Bridging fibrosis with variable lobular disarray
3	Liver cirrhosis with regenerative nodules and extensive fibrosis
Bile duct loss	
0	No bile duct loss
1	Bile duct loss in < 1/3 of portal tracts
2	Bile duct loss in 1/3–2/3 of portal tracts
3	Bile duct loss in > 2/3 of portal tracts
Deposition of orcein positive granules	
0	No deposition of granules
1	Deposition of granules in several periportal hepatocytes in < 1/3 of portal tracts
2	Deposition of granules in a variable periportal hepatocytes in 1/3–2/3 of portal tracts
3	Deposition of granules in many hepatocytes in > 2/3 of portal tracts
STAGING	
Stage	Sum of score: fibrosis, bile duct loss and deposition of orcein-positive granules
1 (no progression)	0
2 (mild progression)	1–3
3 (moderate progression)	4–6
4 (advanced progression)	7–9
Sum of score: bile duct loss and fibrosis	
1 (no progression)	0
2 (mild progression)	1–2
3 (moderate progression)	3–4
4 (advanced progression)	5–6

**Table 3:** Ludwig’s staging system [34]

Ludwig’s staging system	
Stage	Histopathological findings
1	Portal stage: portal oedema, mild portal hepatitis, non-destructive cholangitis, lymphocyte infiltration in bile ducts, ductular proliferation, periductal “onion-skin” fibrosis, fibrous-obliterative cholangitis
2	Periportal stage: periportal fibrosis, interphase hepatitis, portal tracts enlargement
3	Septal stage: bridging fibrous septa, degeneration and disappearance of bile ducts
4	Cirrhosis

### Differential diagnosis

**Fig. 4:**
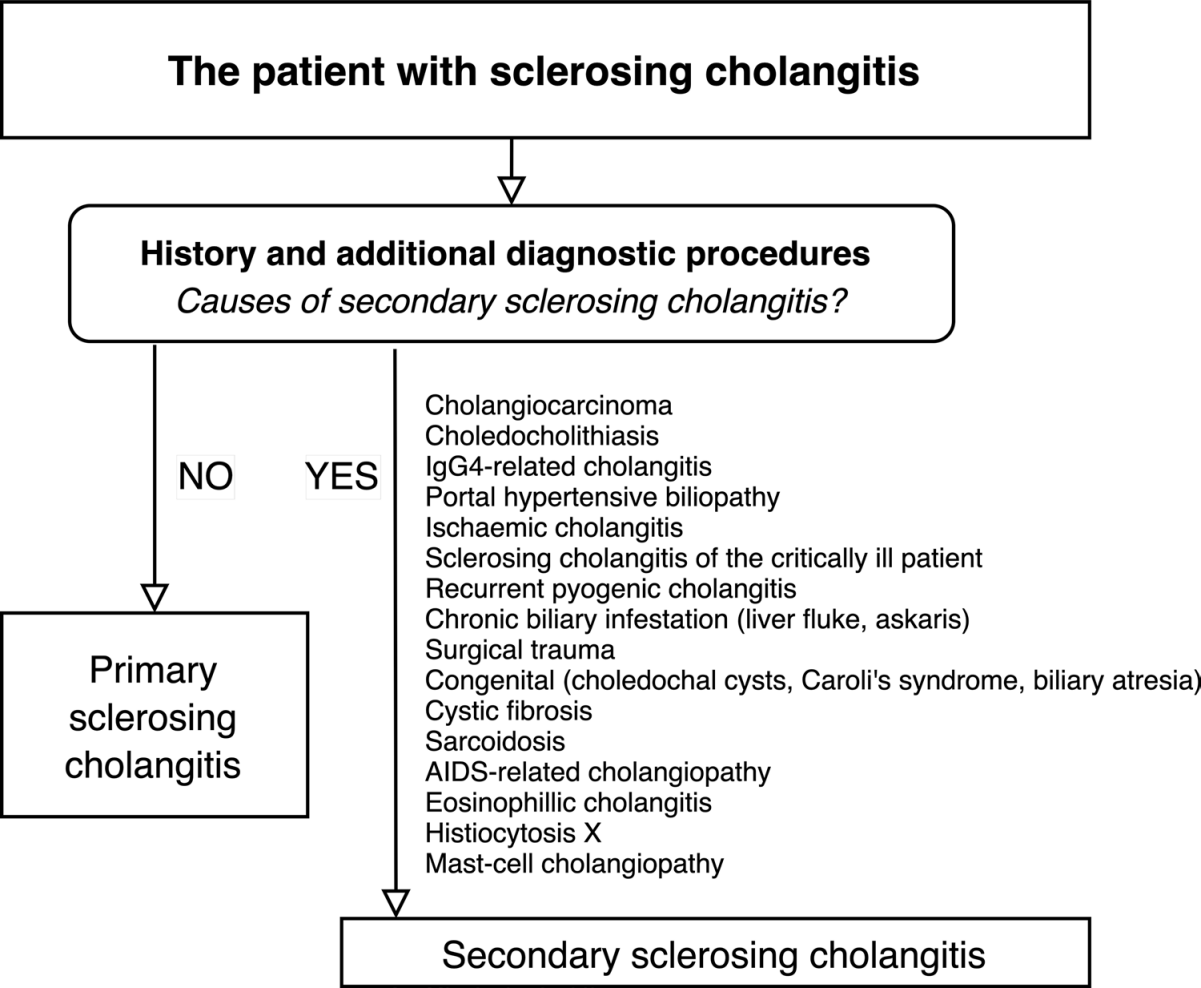
Algorithmic approach to the patient with sclerosing cholangitis. Causes of secondary sclerosing cholangitis have to be excluded before a diagnosis of PSC can be made

The diagnosis of PSC should be made only after secondary causes of sclerosing cholangitis (SSC) have been ruled out [2]. IgG4-related cholangitis (IgG4-RC), which, like PSC, is associated with biliary strictures, elevated serum liver tests, and serum IgG4 and also similar symptoms may be excessively difficult to distinguish. Nevertheless, IgG4-RC is most commonly found in elderly men, often with long-term exposure to potentially harmful chemicals (“blue collar work”). The diagnosis should be made using HISORt criteria, based on histological, imaging, and serological (IgG4, IgG4/IgG1 ratio) findings, other organ involvement (autoimmune pancreatitis, sialadenitis, many others) and response to corticoid treatment [39, 40]. Therefore, patients with PSC should be tested at least once for IgG4 serum levels [29]. For other differential diagnoses see Fig. 4.

## Complications of PSC and their management

### Dominant strictures and bacterial cholangitis

In PSC, persistent biliary inflammation and fibrogenesis lead to a gradual narrowing of bile ducts and the formation of fibrotic strictures. A dominant stricture is observed in around 60% of cases during long-term follow-up and refers to a stenosis visible at cholangiography with a diameter ≤ 1.5 mm in the common bile duct or ≤ 1.0 mm in the hepatic ducts within 2 cm of the main biliary confluence [3, 32]. The development of such strictures is part of the progression of PSC and is associated with a risk of cholangiocarcinoma and reduced transplant-free survival [3]. MRCP should precede ERCP to assess biliary obstruction and detect associated complications [31, 32]. When the progression of known strictures is suspected or a new dominant stricture is identified at imaging, ERCP with ductal brush sampling and fluorescent in situ hybridization (FISH), if available, is recommended to rule out cholangiocarcinoma [32, 41]. Endoscopic treatment of dominant strictures should be performed only in symptomatic cases of clinical or biochemical deterioration [32]. A recent retrospective study, however, suggests the benefit of annual diagnostic ERCP in patients with dominant strictures with endoscopic treatment of strictures also in asymptomatic patients with an impact on transplant-free survival and incidence of recurrent cholangitis [42]. To endoscopically treat dominant strictures, balloon dilation is preferred over stent placement despite similar efficacy, due to the lower incidence of serious adverse events (pancreatitis, bacterial cholangitis) associated with the procedure [43]. Pancreatitis and bacterial cholangitis are the most common post-ERCP complications in PSC. Therefore, administration of NSAIDs (100 mg of diclofenac or indomethacin rectally) and prophylactic antibiotics before the procedure are necessary in all PSC patients [32]. Dominant strictures are often associated with symptoms of bacterial cholangitis, which is a common complication of PSC not only in relation to ERCP. The management depends on the severity of cholangitis. In more severe cases, hospitalization is necessary for intravenous treatment, including broad-spectrum antibiotics (e.g. ceftriaxone, piperacillin-tazobactam, ampicillin-sulbactam) [23]. Patients prone to recurrent cholangitis should have antibiotics available in case they develop symptoms and in some cases, especially when liver transplantation is not available, it is necessary to keep patients with advanced disease on long-term prophylactic rotating antibiotic therapy [1, 13].

### Cholangiocarcinoma

Cholangiocellular carcinoma (CCA) is the most common hepatobiliary malignancy in PSC dramatically worsening the prognosis. It develops in approximately 10–15% of patients over the lifetime, with half of the cases being diagnosed in the first year after diagnosis of PSC probably due to the development of CCA-related symptoms [3]. Hilar localization is most common and often associated with the presence of a dominant stricture [3]. CCA may remain asymptomatic for a long time, but when the symptoms appear and CCA is found, an advanced tumor stage is usually present. CCA screening is therefore an essential part of PSC management. As part of the screening, it is recommended to re-evaluate the clinical status of a patient and the results of laboratory and imaging studies. Annual ultrasound is recommended for analysis of gallbladder (see below), bile ducts, and liver parenchyma [2]. Annual MRI of the liver combined with MRCP, could provide higher sensitivity for the detection of potentially malignant lesions [41]. The use of CA19-9 as a tumor marker is debatable and some guidelines no longer recommend it in CCA screening due to low accuracy, as up to 30% of patients with elevated levels may be false positive [44, 45]. In case of CCA suspicion, ductal sampling (brush cytology or/and endobiliary biopsies) and in equivocal cases also chromosomal assessment using FISH is recommended [32]. Recently, the diagnostic accuracy of brush cytology was found to be higher than cholangioscopy in indeterminate biliary strictures [46]. However, data vary between studies and some, in turn, favor cholangioscopy [47]. Histological assessment should be performed to confirm the diagnosis of CCA and the decision on which method to use should be determined by a multidisciplinary team in a specialized center [45]. Therefore, referring a patient with suspected CCA to such a center is essential.

### Gallbladder carcinoma

Gallbladder diseases are relatively common findings in PSC patients, represented by mainly gallstones or cholecystitis found in 25% of patients [30]. Polyps occur in 10–16% of PSC patients [48, 49], are mostly benign and are associated with malignancy predominantly at sizes greater than 10 mm [49]. The current AGA PSC guideline recommends annual ultrasound screening for gallbladder polyps in all PSC patients (in line with EASL Clinical Practice Guidelines [2]) and cholecystectomy if polyps larger than 8 mm are present [41]. Still, adenocarcinoma can also be found in polyps smaller than 5 mm, therefore cholecystectomy should be considered regardless of the size of the polyp [2, 48]. Nevertheless, short-term pre-cholecystectomy surveillance of polyps without features of high malignancy risk appears beneficial, as up to 80% of polyps are not detected at subsequent imaging [49].

### Cirrhosis and hepatocellular carcinoma

Management of cirrhosis and hepatocellular carcinoma (HCC) screening in PSC patients should not differ from cirrhosis of other etiologies. Use of imaging modalities is recommended for HCC surveillance and testing of alpha-fetoprotein (AFP) levels may be also considered [41]. However, a retrospective study of cirrhotic PSC patients did not detect a single case of HCC [50]. Complications of cirrhosis should be managed according to standard guidelines [51].

### Liver transplantation

Liver transplantation is the only curative treatment and life-saving intervention in PSC. Several specific indications for liver transplantation are defined for PSC, including recurring uncontrollable cholangitis, decompensated secondary biliary cirrhosis, and similarly to PBC, intractable pruritus may also be an indication [27, 52]. Liver transplantation prolongs the survival of a recipient by more than 10 years in 70–80% of cases [53, 54]. Nevertheless, in around 20%, recurrent PSC (rePSC) occurs in less than 5 years after liver transplantation (Table 4) with a negative impact on patient survival [53, 54]. Risk factors for rePSC are younger age and the presence of UC [54]. It is important to distinguish between rePSC and post-transplant biliary strictures, which can also occur, even more often (36%), for various reasons (ischemia, infection, treatment induction) but similarly with a negative impact on survival [53]. Two techniques, Roux-en-Y choledochojejunostomy, and duct-to-duct anastomosis are used in biliary reconstruction in liver transplantation in PSC patients. They are equivalent in terms of survival, incidence of biliary strictures, recurrent PSC and cholangiocarcinoma [55], but duct-to-duct anastomosis has been shown to have a lower incidence of ascending cholangitis and should therefore be considered as the method of choice [56].

**Table 4:** Large-sized (*n* > 100) case-series on liver transplantation in PSC with recurrence and patient survival rates

Study	Type of study	Years	No. of patients	Recurrence (%)	Patient survival after 5 years (%)
Goss et al*.* [80]	Single center	1984–1996	127	8.6	85
Alabraba et al*.* [81]	Single center	1986–2006	230	23.5	68
Campsen et al*.* [82]	Single center	1988–2006	130	16.9	84
Hildebrand et al*.* [53]	Multicenter	1990–2006	305	20.3	84.8
Ravikumar et al*.* [54]	Multicenter	1990–2010	565	14.3	79
Lindstrom et al*.* [83]	Multicenter	1984–2007	440	19	73
Gordon et al*.* [84]^a^	Multicenter	1998–2013	307	11	82.5

^a^Living donor liver transplantation study

### Pruritus

Pruritus is a concomitant manifestation of cholestatic disease and is experienced by a large group of PSC patients during the course of the disease, although the intensity may vary [24]. The molecular pathogenesis of cholestatic pruritus has not been elucidated, which is also reflected in the frequent lack of drug control in some cases leading to liver transplantation. In PSC, dominant strictures should be excluded as a cause of pruritus. The so-far recommended first-line medical treatment is cholestyramine (4–16 g/day, administered separately from other drugs). In case of its ineffectiveness or intolerance, rifampicin, naltrexone, and sertraline may be considered as the following steps [2, 24]. Results of the most recently published randomized, placebo-controlled FITCH trial (‘fibrates for cholestasis-associated itch’) clearly showed the efficacy of bezafibrate (400 mg/day) in the treatment of severe or moderate cholestasis-associated pruritus in PSC and PBC [57]. Thus, bezafibrate may become the 1st line medical treatment of pruritus in PSC in the future (Table 5).

**Table 5:** Medications used in the management of PSC and its complications

Indication	Drugs
PSC	Ursodeoxycholic acid (UDCA): 15–20 mg/kg/day
Cholangitis	Antibiotics: in the pocket or prophylactic rotating ATBs
Pruritus	Bezafibrate, cholestyramine, rifampicin, naltrexone, sertraline
Autoimmune hepatitis	Predniso(lo)ne or budesonide, azathioprine
IBD	5-ASA, corticosteroids, biologics

*PSC *primary sclerosing cholangitis, *IBD *inflammatory bowel disease, *5-ASA *5-aminosalicylic acid, *ATBs *antibiotics

### IBD and colorectal carcinoma

Concomitant IBD is the dominant finding present in more than two-thirds of PSC patients [8]. Regular ileocolonoscopic screening with segmental biopsies is therefore recommended, first at the time of PSC diagnosis and then, when negative, at least every 5 years [32]. Colonic involvement is characteristic, regardless of whether ulcerative colitis, Crohn's disease or IBD-unspecified (75%, 21%, and 4%, respectively; *n* = 579) is diagnosed [8]. PSC-IBD patients have an increased risk of colorectal carcinoma (CRC), which can develop much sooner than in IBD alone [58], but also higher risk of hepatobiliary malignancies and death [7, 22, 58]. PSC and PSC-IBD patients under regular surveillance have better outcomes [7, 58]. Therefore, screening for malignancies by annual ileocolonoscopy including chromoendoscopy and histological sampling is strongly recommended [32]. Suspicious lesions should be endoscopically resected. Proctocolectomy should be considered if high-grade dysplasia is unraveled [32]. Treatment of the underlying PSC-IBD should be adjusted to the prevailing phenotype [8].

### Pharmacological management of PSC

**Fig. 5:**
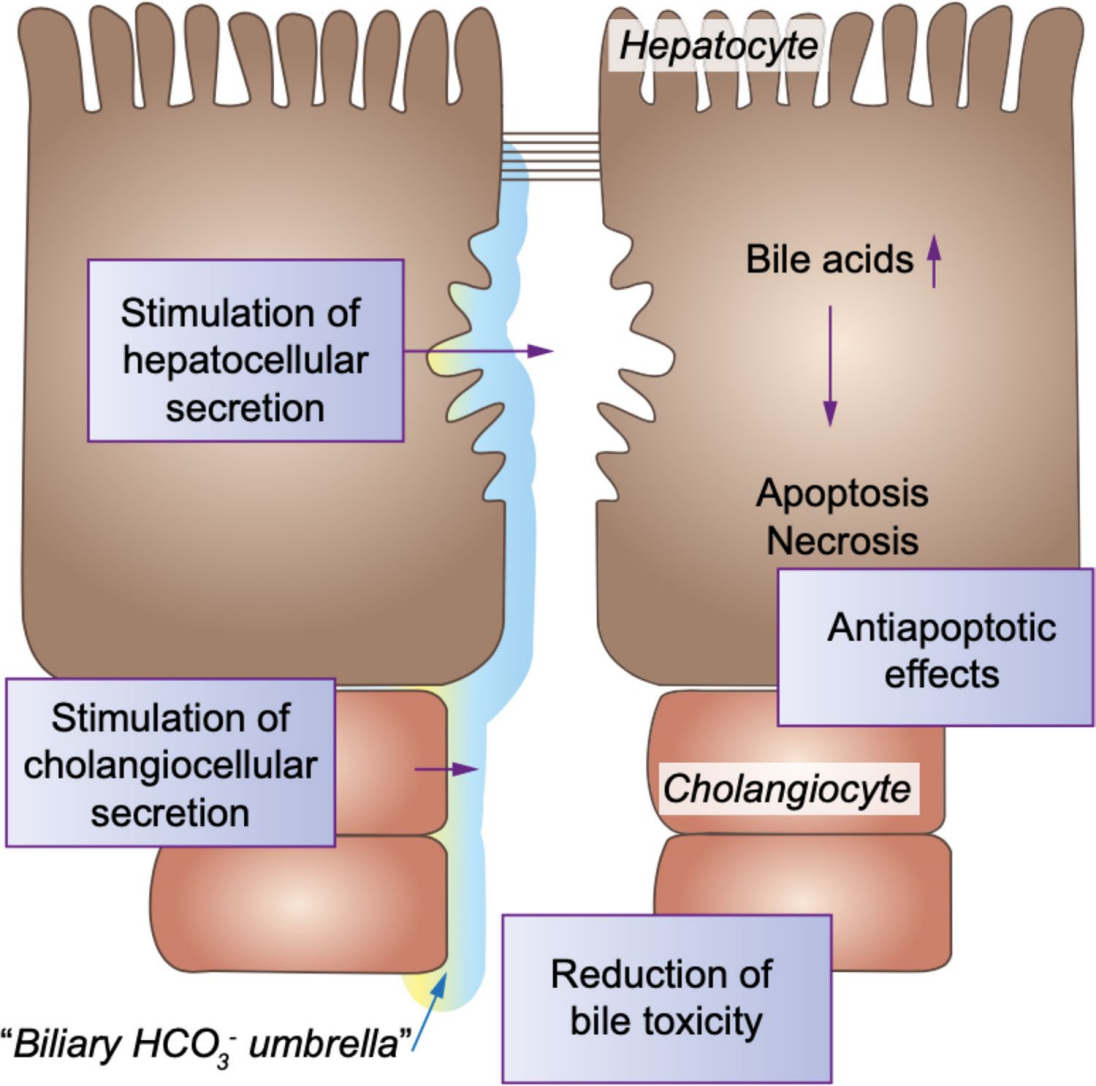
Major mechanisms and sites of action of UDCA in cholestatic diseases [60]. Reprinted with permission from the author and Elsevier

Ursodeoxycholic acid (UDCA), a hydrophilic human bile acid, represents the first-line therapy in the treatment of PBC (13–15 mg/kg/day) where it leads to improved transplant-free survival in all patients under study (*n* = 3902) according to recent analysis and is associated with normal life expectancy as monotherapy in up to two-thirds of PBC patients treated [59]. In PSC and other very rare chronic cholestatic diseases, much less data are available on long-term treatment with UDCA, but due to its potent anticholestatic effects and its excellent safety profile when administered at moderate doses of 13–20 mg/kg/day, UDCA is widely prescribed at least in Continental Europe where the so far best LTx-free survival data for patients with PSC have been reported [7, 27, 60]. UDCA exerts protective effects in the hepatobiliary tract, mainly by posttranscriptional stimulation of hepatobiliary secretion of bile acids, organic anions, and bicarbonate, by which UDCA contributes to the stabilization of the “biliary bicarbonate umbrella”, a protective molecular mechanism at the level of the bile ducts, but also reduces bile toxicity and has anti-apoptotic and anti-inflammatory effects (Fig. 5) [61]. Nevertheless, opinions differ on the role of UDCA in the treatment of PSC, mainly due to the fact that despite a marked improvement of serum markers of cholestasis [62, 63], no significant improvement of transplant-free survival was found in UDCA-treated patients in this study endpoint underpowered trials with low (13–15 mg/kg; *n* = 102), moderate (17–23 mg/kg; *n* = 198) or very high daily doses (28–30 mg/kg, *n* = 150), when compared to placebo [64–66]. Conversely, very high doses of UDCA (28–30 mg/kg) were potentially harmful, leading to an increase in adverse events such as the development of varices or listing for liver transplantation and are generally not recommended in PSC [2, 29, 66]. In moderate doses (15–20 mg/kg) UDCA may exert protective effects in the hepatobiliary tract, but its effectiveness as monotherapy is probably not sufficient to prevent PSC progression in a majority of patients. Nevertheless, discontinuation of UDCA has been shown to cause worsening of symptoms, of serum liver tests and of the Mayo Risk Score and should therefore be well justified in patients stable on therapy [67].

24-Norursodeoxycholic acid (*nor*UDCA) is a side chain-shortened UDCA homologue that is conjugated only at very limited rates and can be passively absorbed by cholangiocytes and undergo cholehepatic shunting with the induction of bicarbonate-rich choleresis again strengthening the biliary bicarbonate umbrella, but anti-inflammatory, anti-lipotoxic, anti-fibrotic and anti-proliferative effects were also proposed [61]. In a phase II clinical trial [68], *nor*UDCA dose-dependently reduced serum ALP, GGT, AST, and ALT levels. A multicenter phase III trial is now ongoing with a focus on endpoints such as histological progression and change in ALP levels (NCT03872921) (Table 6).

**Table 6:** Medications under evaluation in randomized, placebo-controlled trials for the treatment of PSC

Drug	Mechanism of action	Phase II results
*nor*UDCA	induction of bicarbonate-rich choleresis, biliary HCO_3_^−^ “umbrella” strengthening	dose-dependent reduction of ALP, GGT, AST and ALT
Obeticholic acid	FXR agonism (steroidal)	dose-dependent reduction of ALP, no change in fibrosis markers
Cilofexor	FXR agonism (non-steroidal)	dose-dependent improvement of ALP, transaminases and markers of fibrosis
Aldafermin	FGF19 analogue: down-regulation of bile acid synthesis	reduction of serum transaminases and fibrogenesis markers, no effect on ALP
Bezafibrate	PPAR agonism: reduction of bile acid synthesis and increased phospholipid secretion	reduction of ALP improvement of pruritus
Vancomycin	modulation of the intestinal microbiota, anti-inflammatory effects	ALP reduction, Mayo Risk Score decline, symptoms decrease

Obeticholic acid (OCA), a semi-synthetic 6-ethyl analogue of chenodeoxycholic acid, acts as a potent FXR agonist affecting bile acid synthesis, inflammation, and liver fibrosis [61]. OCA has recently been approved as a second-line add-on therapy to UDCA in PBC, due to evidence of biochemical efficacy in a phase III study [60]. In PSC, a phase II clinical trial demonstrated reduced ALP levels in the group receiving a 5–10 mg dose of OCA, but no change in fibrosis markers [69]. Unfortunately, dose-dependent pruritus as a side effect described in both PBC and PSC may lead to reduced patient compliance with OCA treatment as was seen in these trials. OCA induces endogenous FGF19 synthesis, the proliferative properties of which have been shown in experimental animals to pose a risk at higher FGF19 serum levels of developing hepatobiliary malignancies. This aspect needs careful evaluation for hepatobiliary malignancy when OCA treatment is further studied in PSC (Table 6).

Cilofexor, a nonsteroidal FXR agonist, led to a dose-dependent improvement of serum markers of cholestasis, transaminases, and markers of fibrosis in a recent phase II trial [70]. A phase III clinical trial is now ongoing with a progression of fibrosis after 8 months of treatment being the primary outcome measure (NCT03890120). As for OCA, the proliferative effects of FGF19 may need particular attention during long-term treatment (Table 6).

Fibroblast growth factor 19 (FGF19), product of the FXR target gene in the ileocyte, is responsible for much of the FXR-mediated hepatic effects in the regulation of bile acid homeostasis, particularly by down-regulation of CYP7A1, a rate-limiting enzyme in hepatic bile acid synthesis. Its engineered non-tumorigenic FGF19 analogue, NGM282 (aldafermin) resulted in a phase II trial to decrease in serum transaminases and robust reduction of markers of fibrogenesis (ELF score, pro-C3), but had no effect on serum markers of cholestasis such as ALP levels [71]. Markers of fibrosis next to markers of cholestasis are widely regarded as biomarkers of PSC survival [72]. Future longer-term studies should also include cholangiographic and elastographic changes (Table 6).

#### Fibrates

Targeting nuclear receptors in the treatment of cholestatic diseases seems to be a suitable choice, not only through FXR, but also by activation of peroxisome proliferator-activated receptors (PPAR). Fibrates, commonly used in the treatment of hypertriglyceridemia, act as PPAR-α and PPAR-δ agonists in the liver through which they mediate anti-inflammatory effects and also lead to a reduction in bile acid synthesis and increased phospholipid secretion [61]. In PSC patients, bezafibrate markedly improved serum ALP levels in cohort studies and is now investigated in a phase III trial (NCT04309773) [73]. Severe to moderate pruritus was clearly improved by bezafibrate in patients with PSC as mentioned above [57].

#### Antibiotics

Modulation of the intestinal microbiota is becoming an increasingly relevant topic, which is also supported by the effectiveness of long-term antibiotic treatment with metronidazole and vancomycin on biochemical parameters, Mayo risk score, and symptoms in PSC [74, 75]. Therefore, the correct setting of chronic low-dose antibiotic treatment with regard to the possible development of antibiotic resistance could represent a potential treatment option in the future, especially in patients with frequent episodes of recurrent cholangitis. Vancomycin is now evaluated in phase III (NCT03710122). In some patients, fecal transplantation may also be an interesting treatment option as reported recently [76].

### Prognosis

Various prognostic scores have been developed until the recent past to adequately predict the individual prognosis for patients with PSC. Next to the well-established Mayo risk score for PSC, the most promising new scores include the Amsterdam-Oxford score and the Primary sclerosing cholangitis risk estimate tool (PREsTo) (Table 7).

**Table 7:** Prognostic scores used for PSC

Prognostic score	Included markers	Link
Mayo risk score (revised) [85]	bilirubin, albumin, AST, age, variceal bleeding	http://www.psc-literature.org/mrscalc.htm
Amsterdam-Oxford score [86]	bilirubin, albumin, ALP, AST, platelets, PSC subtype, age at diagnosis	http://www.fcbkapp.nl/psc/8/
PREsTo [87]	bilirubin, albumin, ALP, AST, platelets, hemoglobin, sodium, age, years since diagnosis	https://rtools.mayo.edu/PRESTO_calculator/

*PREsTo *Primary sclerosing cholangitis risk estimate tool, *ULN *upper limit of normal

## Conclusion

**Fig. 6:**
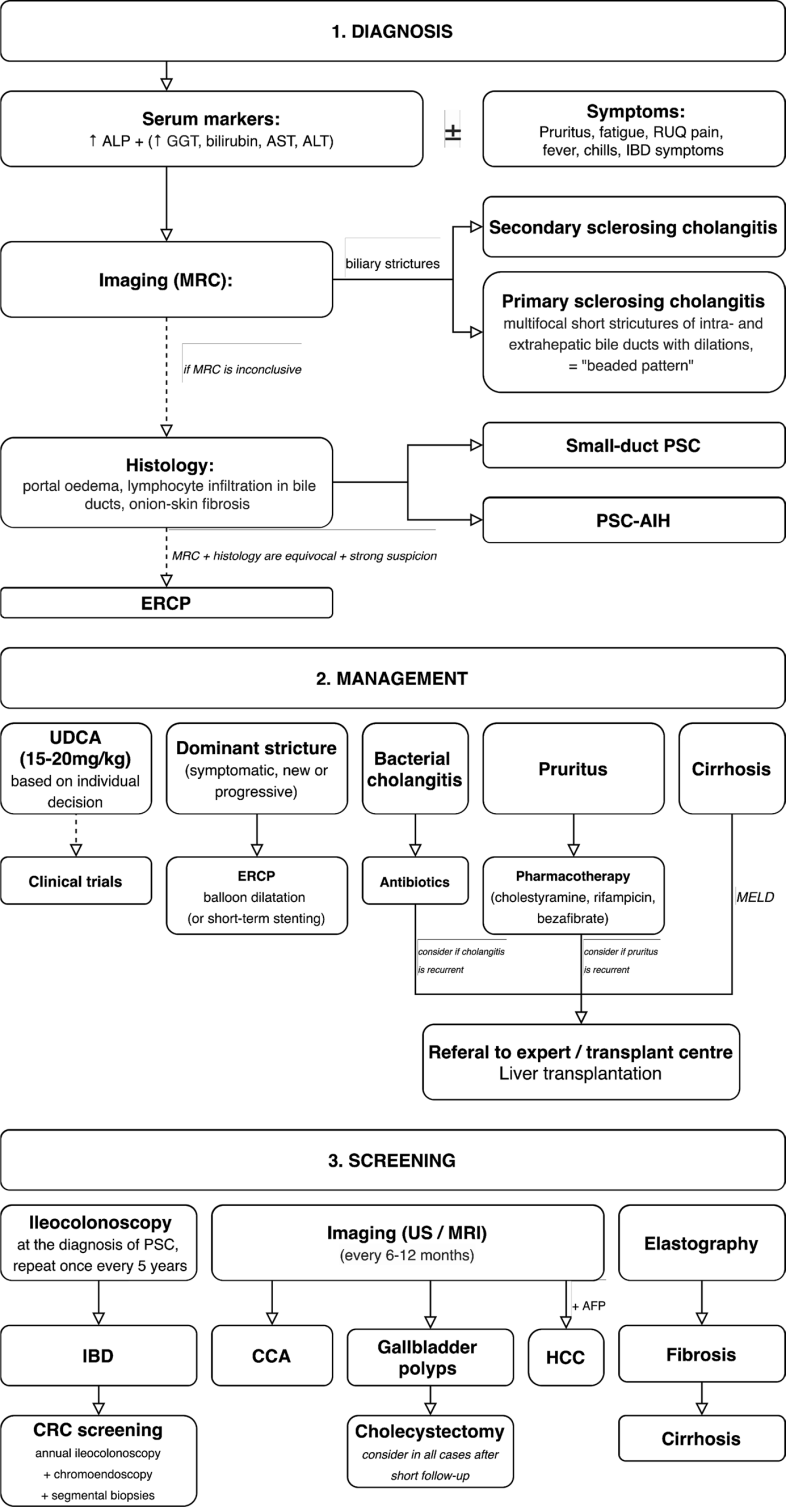
Algorithmic approach to (1) diagnosis and (2) management of PSC and its complications, and (3) recommended screening for patients with PSC

Primary sclerosing cholangitis is a complex, incompletely unraveled, chronic, and progressive hepatobiliary disease. Patients may benefit from an algorithmic diagnostic and therapeutic approach for their best possible care (Fig. 6). Elucidation of the disease pathogenesis, optimization of early diagnosis, follow-up, and screening for malignancies and their early treatment, as well as the development of effective combination therapies for PSC including anticholestatic, anti-inflammatory, and antifibrotic compounds to halt disease progression and improve patient survival, are major needs and challenges for the near future.
